# Supplementary material for: Mutations in the Staphylococcus aureus Global Regulator CodY Confer Tolerance to an Interspecies Redox-Active Antimicrobial
Source: bioRxiv. 2024 Jul 3:2024.07.02.601769. Preprint. [Version 1] doi: 10.1101/2024.07.02.601769 (PMC11261909; doi:10.1101/2024.07.02.601769)
Supplement: Supplement 1 [file media-1.pdf]

SUPPLEMENTARY FIGURES

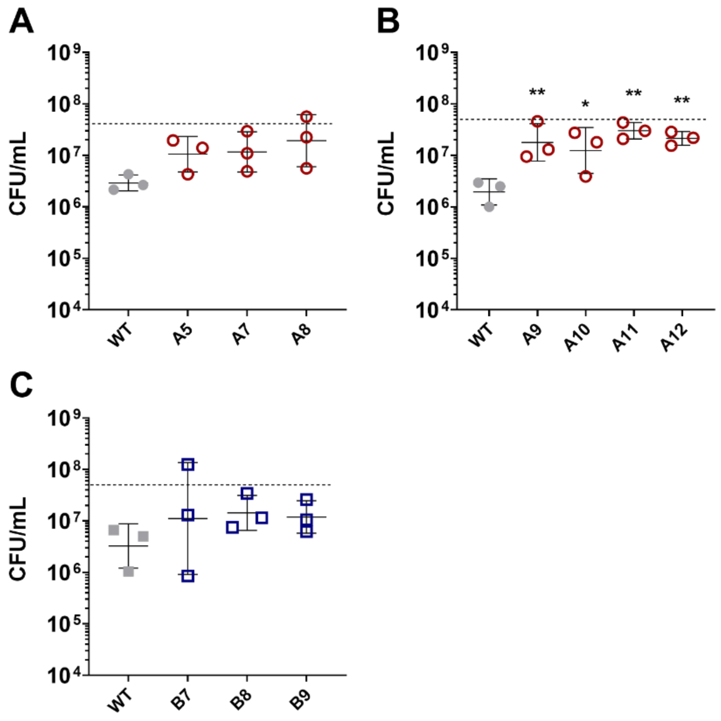

Supplemental Figure 1. PYO tolerance of experimental evolution isolates. PYO tolerance of terminal isolates from population A (**A, B**) and population B (**C**). Values indicate *S. aureus* viable cell counts after 20 hours of treatment with 200 µM PYO. The dashed lines indicate the mean initial cell density (CFU/mL) at the time of PYO addition. Data shown are the geometric mean ± geometric standard deviation of three biological replicates. Significance is indicated for comparison to the parent strain as determined by a one-way ANOVA using Dunnett's correction for multiple comparisons. ($^{*}P < 0.05$, $^{**}P < 0.01$).

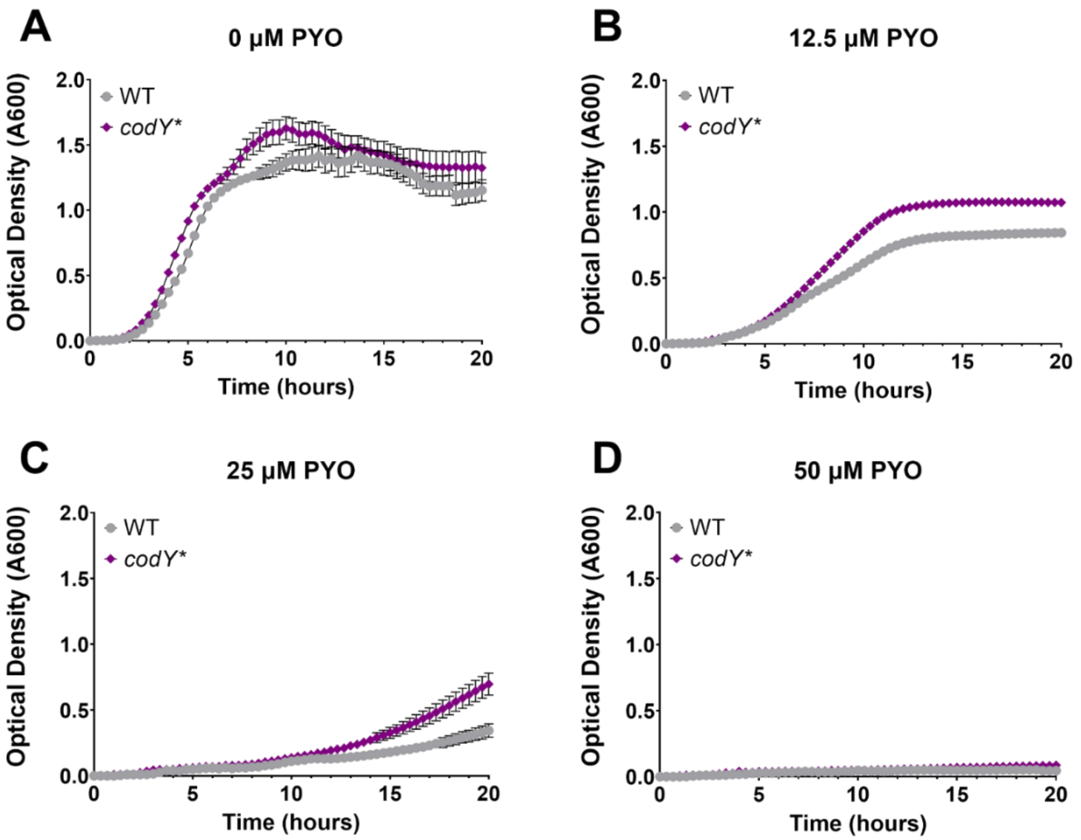

Supplemental Figure 2. Growth of the *codY mutant in low concentrations of PYO is modestly greater than WT**. Growth curves shown as OD_{600} measurements of the WT and the *codY** mutant in M63 containing (**A**) 0 µM PYO (DMSO control), (**B**) 12.5 µM PYO, (**C**) 25 µM PYO, and (**D**) 50 µM PYO. Data shown are the mean ± standard error of seven biological replicates.

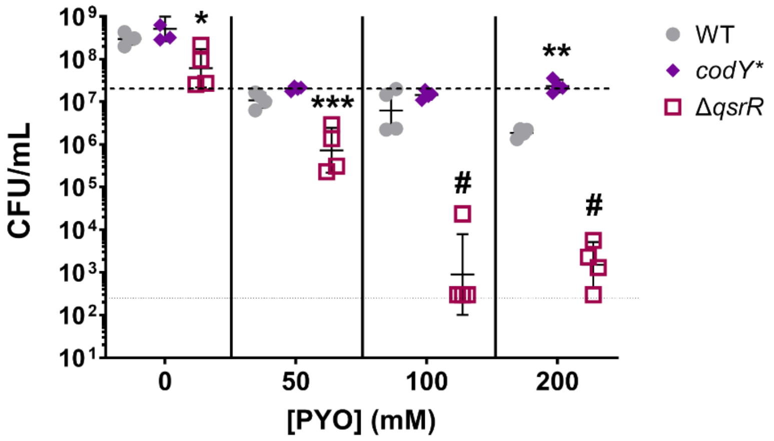

Supplemental Figure 3. A Δ*qsrR* mutant exhibits reduced tolerance to PYO. Viable cell counts are shown for the WT, and the *codY** and Δ*qsrR* mutants after 20-hour treatment with the indicated concentration of PYO. Data shown are the geometric mean ± geometric standard deviation of four biological replicates. The upper, dashed line indicates the mean initial cell density (CFU/mL) for all strains and the lower, dotted line indicates the limit of detection. Significance is shown for comparisons to the respective WT condition, as determined by a two-way ANOVA using Dunnett's correction for multiple comparisons. ($^{*}P < 0.05$, $^{**}P < 0.01$, $^{***}P < 0.001$, $^{\#}P < 0.0001$).

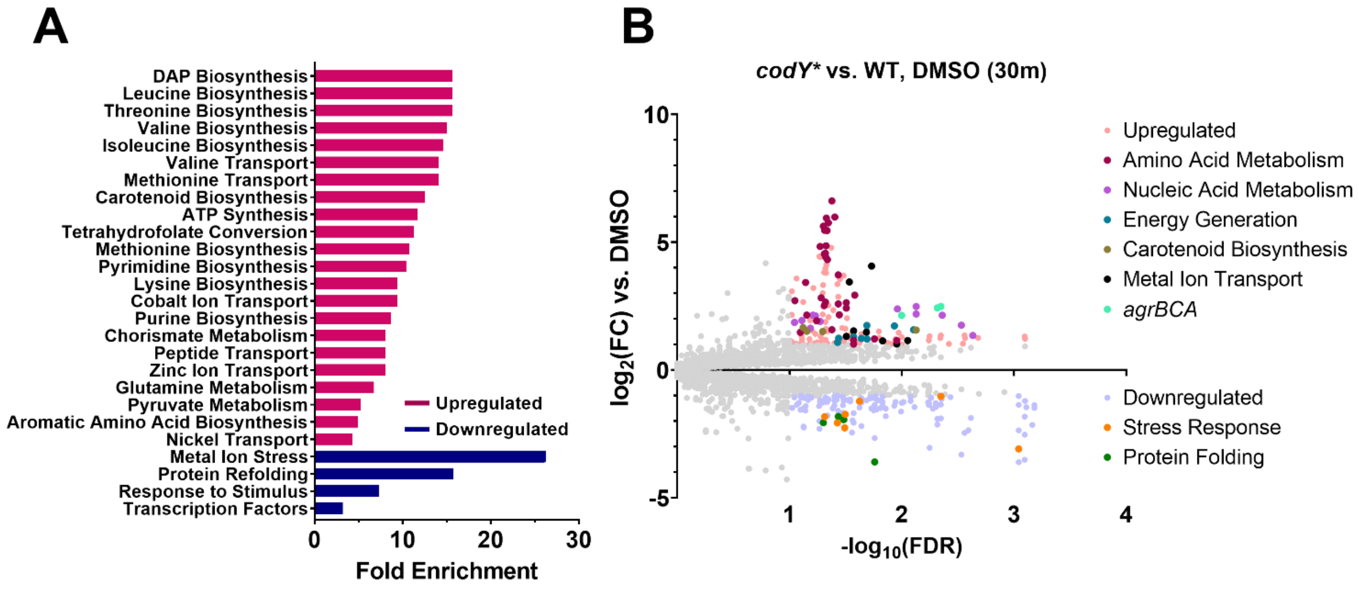

Supplemental Figure 4. The transcriptional profile of the *codY mutant compared to WT is consistent with loss of CodY activity**. Differential gene expression of *codY** compared to WT after 30 minutes of incubation in DMSO. (**A**) Enriched GO pathways from upregulated and downregulated differentially expressed genes. (**B**) Volcano plot of $\log_2$(fold change gene expression) and -log10(false discovery rate). Upregulated genes are shown in light red and downregulated genes are shown in light blue. Individual genes from several pathways in (**A**) are further highlighted. A list of genes included in each pathway can be found in **Supplementary Data File 03**.

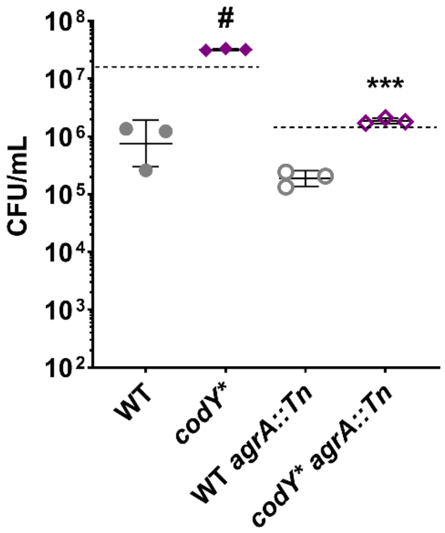

Supplemental Figure 5. Increased PYO tolerance conferred by the *codY mutation does not require *agr* activity**. Viable cells counts of WT, the *codY** mutant, WT *agrA*::Tn, and a *codY** *agrA*::Tn double mutant after 20-hour treatment with 200 μM PYO. Dashed lines indicate the approximate CFU at the time of PYO addition for the indicated strains. Data shown are the geometric mean ± geometric standard deviation of three biological replicates. The dashed lines indicate the mean initial cell density (CFU/mL) for the strains they overlap. Significance is shown for a comparison to the respective WT background and was determined by a one-way ANOVA using Šídák's correction for multiple comparisons. ($^{***}P < 0.001$, $^{\#}P < 0.0001$).

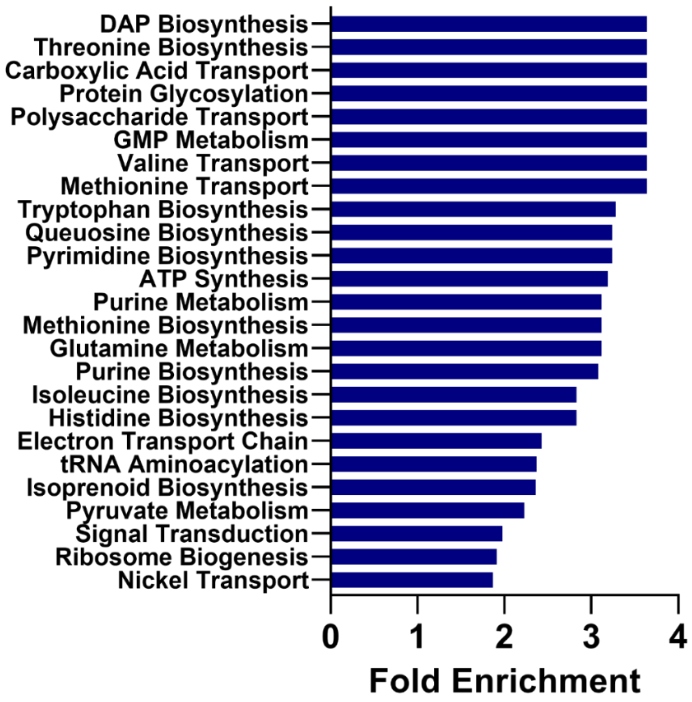

Supplemental Figure 6. Enriched pathways among the downregulated genes in the WT response to PYO after 120 minutes overlap significantly with that of the *codY regulon**. All enriched pathways among downregulated genes in the WT response to PYO compared to WT in DMSO after 120 minutes.

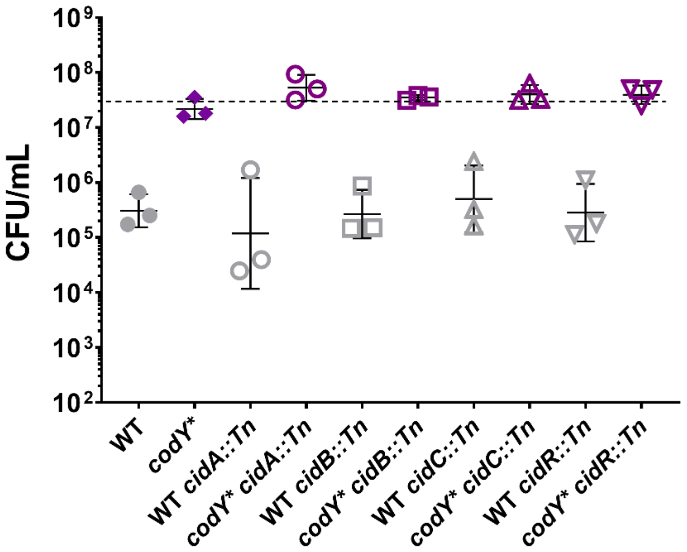

Supplemental Figure 7. Mutations in the *cidABCR* operon do not impact PYO survival in either the WT or *codY backgrounds**. Viable cell counts of WT, the *codY** mutant, and their respective *cid* transposon mutants after 20-hour treatment with 200 μM PYO. Data shown are the geometric mean ± geometric standard deviation of three biological replicates. The dashed line indicates the mean initial cell density (CFU/mL) for all strains. Significance was determined relative to the isogenic parental (WT or *codY**) strain by a two-way ANOVA using Dunnett's correction for multiple comparisons.

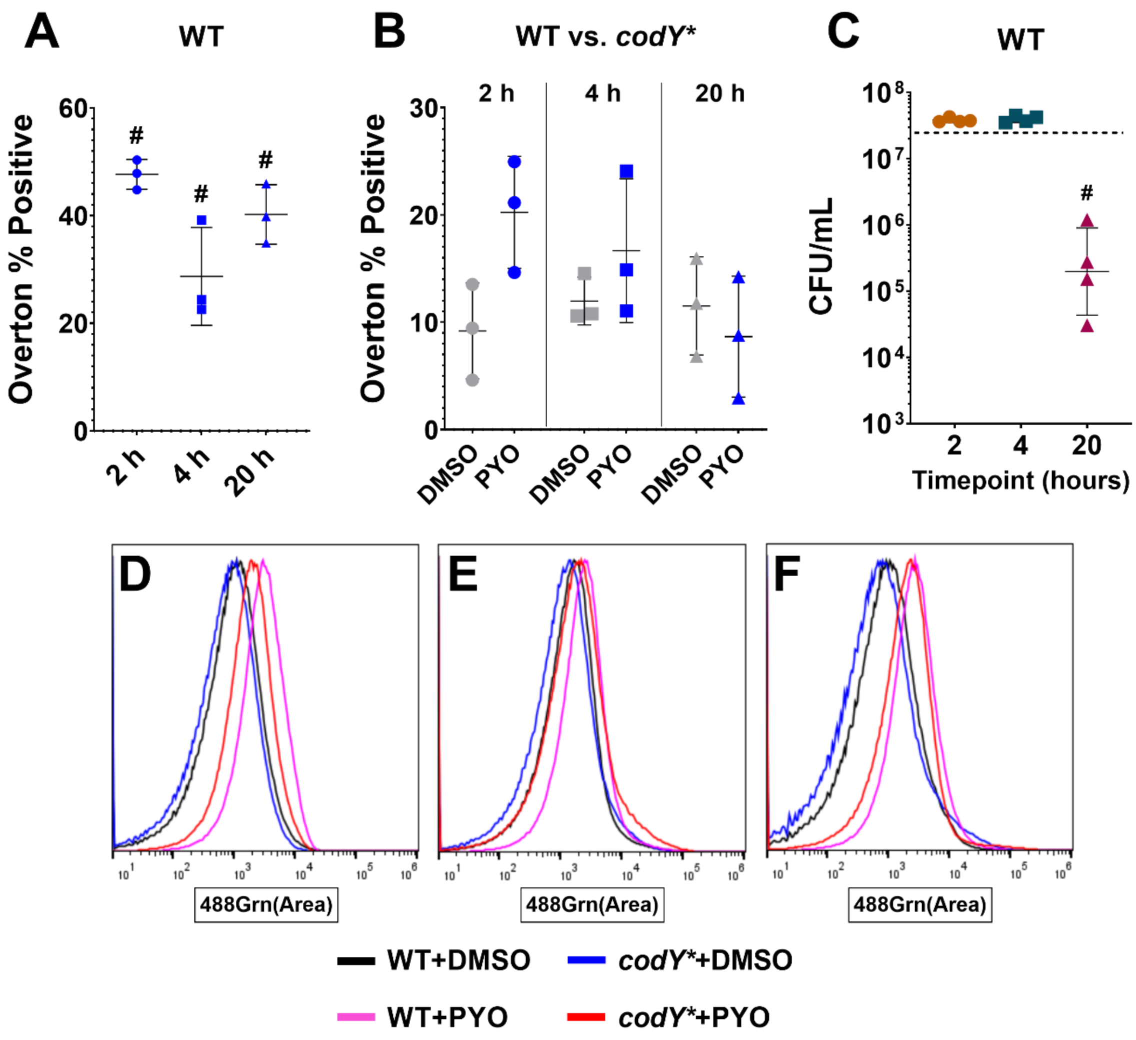

Supplemental Figure 8. PYO induces DNA damage in *S. aureus*. (**A**) Overton % positivity (reflecting the % of the population that has significant fluorescence compared to the control condition) after 2-, 4-, and 20-hour treatment with 200 μM PYO. (**B**) The difference in Overton % positivity between the WT and *codY** mutant in DMSO or PYO after the indicated treatment time. (**A, B**) Data shown are the mean ± standard deviation of three biological replicates. (**C**) Viable cell counts of WT after treatment with 200 μM PYO for the indicated time. Data shown are the geometric mean ± geometric standard deviation of four biological replicates. The dashed line indicates the mean initial cell density (CFU/mL). Significance is shown relative to (**A**) 0% Overton positivity, (**B**) the respective DMSO condition, or (**C**) the initial (0h) cell density as tested by a (**A,**

B) two-way ANOVA using (**A**) Tukey's or (**B**) Šídák's correction for multiple comparisons, or (**C**) one-way ANOVA using Dunnett's correction for multiple comparisons ($^{\#}P < 0.0001$). (**D-F**) Representative histograms from the data underlying (**B**) after (**D**) 2-, (**E**) 4-, and (**F**) 20-hour treatment.

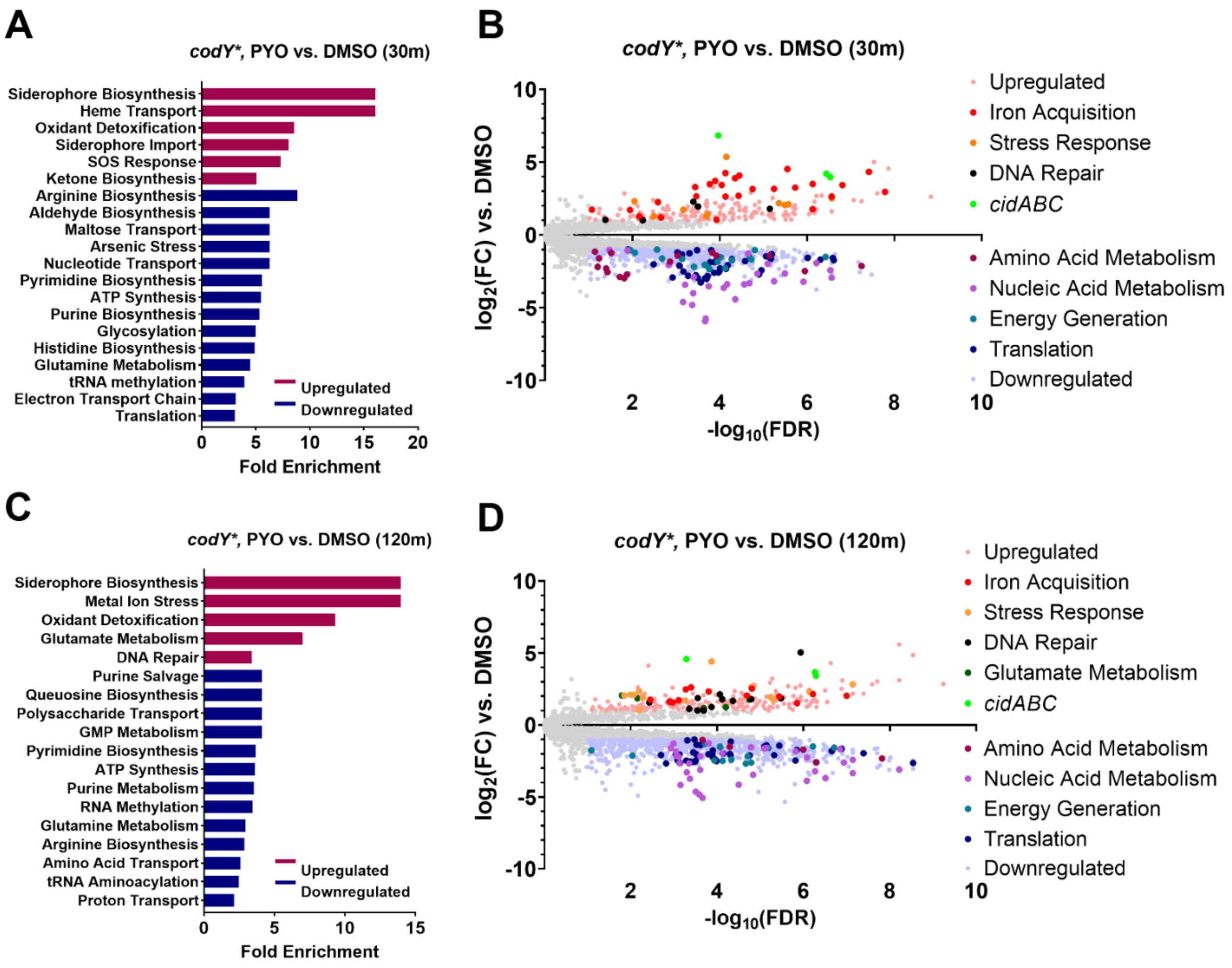

Supplemental Figure 9. The response to PYO of the *codY mutant is similar to that of the WT but shows greater metabolic suppression**. Transcriptional response of the *codY** mutant to PYO compared to the DMSO control after (**A**, **B**) 30 and (**C**, **D**) 120 minutes. (**A**, **C**) Enriched GO pathways from upregulated and downregulated genes. (**B**, **D**) Volcano plot of log_2(fold change gene expression) and -log10(false discovery rate). Highlighted genes comprise the pathways indicated in the figure legend. A list of genes in each pathway can be found in **Supplementary Data File 03**.

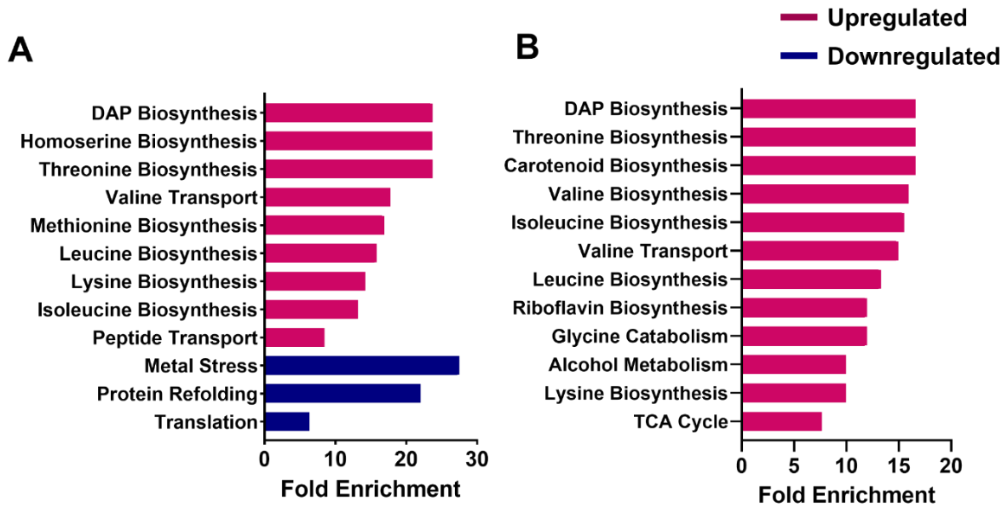

Supplemental Figure 10. Pathways enriched within genes differentially expressed in the *codY mutant compared to the WT in response to PYO are predominantly involved in amino acid metabolism**. Enriched GO pathways from upregulated and downregulated genes in the *codY** response to PYO compared to the WT response to PYO after 30 (**A**) and 120 (**B**) minutes of treatment with PYO.

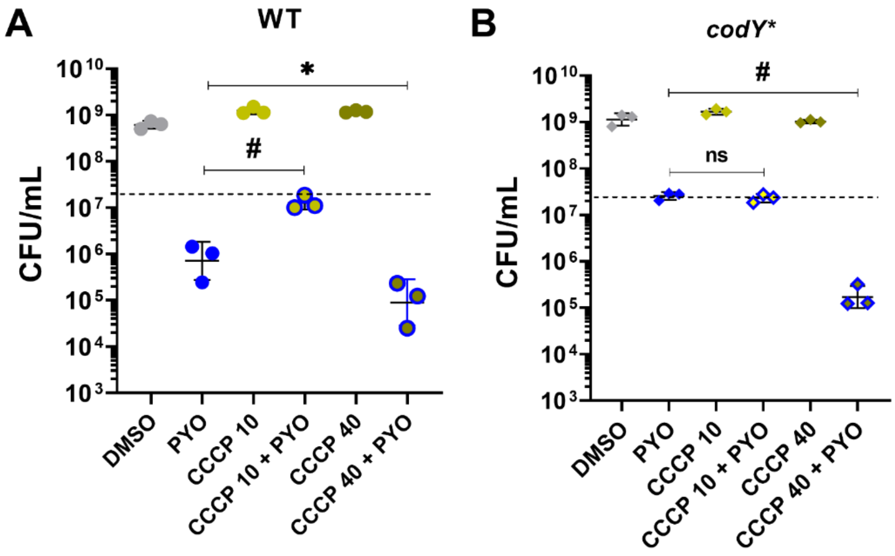

Supplemental Figure 11. Excess CCCP sensitizes both WT and the *codY mutant to PYO**. Effect of CCCP addition (10 μM or 40μM) on survival after 20-hour treatment with either DMSO or 200 μM PYO for the (**A**) WT and (**B**) *codY** mutant. Shown are the viable cell counts. (**A, B**) Data shown are the geometric mean ± geometric standard deviation of three biological replicates. Dashed lines indicate the mean initial cell density (CFU/mL) for all strains. Significance is shown for comparison to the indicated conditions and was determined by a one-way ANOVA using Tukey's correction for multiple comparisons. ($^{*}P < 0.05$, $^{\#}P < 0.0001$).

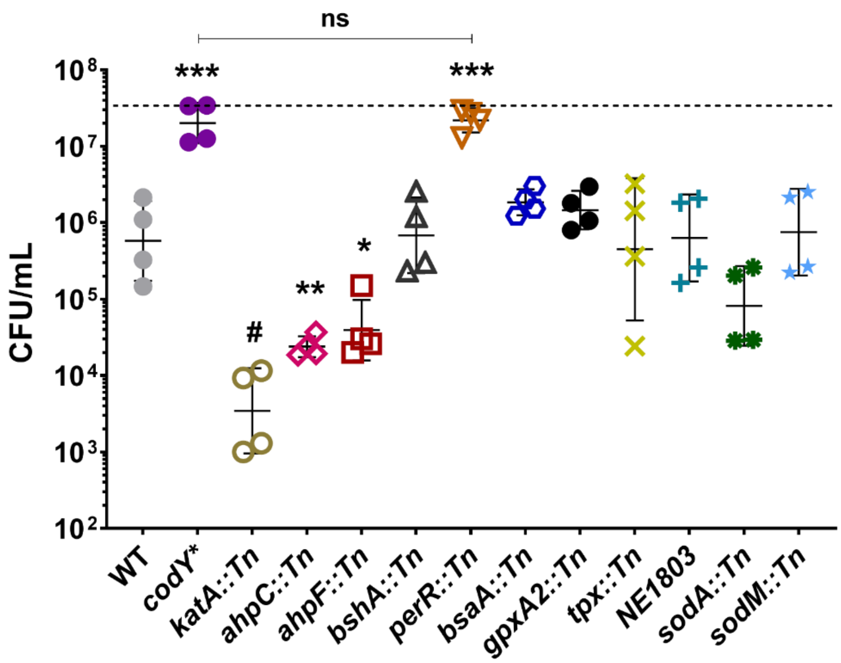

Supplemental Figure 12. Mutants deficient in the response to hydrogen peroxide stress are more sensitive to PYO while a mutant with a constitutive stress response phenocopies *codY**. Viable cell counts of WT, the *codY** mutant, or the indicated transposon mutant strains after 20-hour treatment with 200 μM PYO. The dashed line indicates the mean initial cell density (CFU/mL) for all strains at the time of PYO addition. Data shown are the geometric mean ± geometric standard deviation of four biological replicates. Significance is indicated either for comparisons to the WT or between the indicated strains as determined by a one-way ANOVA using Dunnett's correction for multiple comparisons. ($^{*}P < 0.05$, $^{**}P < 0.01$, $^{***}P < 0.001$, $^{\#}P < 0.0001$).

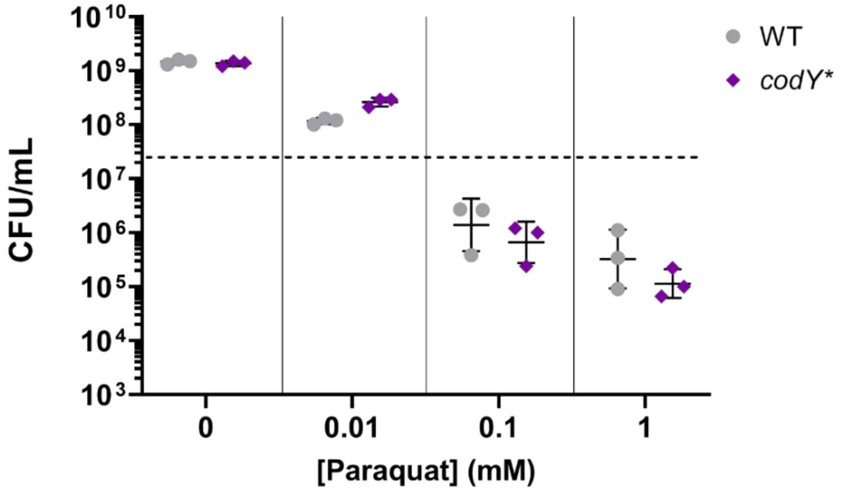

Supplemental Figure 13. WT and the *codY mutant are similarly susceptible to paraquat-derived superoxide**. Viable cell counts of WT and the *codY** mutant after 20-hour treatment with the indicated concentration of paraquat. Data shown are the geometric mean ± geometric standard deviation of three biological replicates. The dashed line indicates the mean initial cell density (CFU/mL) for all strains. Significance was determined for comparison to the respective WT conditions by a two-way ANOVA using Šídák's correction for multiple comparisons.

SUPPLEMENTARY TABLES

Supplemental Table 1. CodY-associated mutations in PYO-evolved isolates.

Designation	Population	Isolate	CodY Mutation
		**Population A**	
200.5-A-1	A	1	Intergenic (+15/-9)<br>AG → GT
200.5-A-2	A	2	Intergenic (+15/-9)<br>AG → GT
200.5-A-3	A	3	R222C
200.5-A-4	A	4	S178L
200.5-A-5	A	5	T125I
200.5-A-7	A	7	R61K
200.5-A-8	A	8	Intergenic (+15/-9)<br>AG → GT
200.5-A-9	A	9	R222C
200.5-A-10	A	10	Intergenic (+15/-9)<br>AG → GT
200.5-A-11	A	11	Intergenic (+15/-9)<br>AG → GT
200.5-A-12	A	12	Y75C
		**Population B**	
200.7-B-1	B	1	G118D
200.7-B-3	B	3	Intergenic (+16/-9)<br>G → T
200.7-B-4	B	4	G118D
200.7-B-5	B	5	G118D
200.7-B-7	B	7	ATG → ATA
200.7-B-8	B	8	G118D
200.7-B-9	B	9	G118D

Supplemental Table 2. Strains and plasmids used in this study.

Name	Description	Source
**Strains**		
Staphylococcus aureus		
JE2	Plasmid-cured derivative of the USA300 strain, LAC	(1, 2)
RN4220	Restriction-deficient mutant of NCTC8325-4	(3)
SB523	JE2 *codY*^{R222C}	This study
NE1555	*codY*::Tn	(2)
SB524	JE2 Δ*qsrR*	This study
NE1532	*agrA*::Tn	(2)
SB525	*agrA*::Tn *codY*^{R222C}	This study
NE1366	*katA*::Tn	(2)
SB526	*katA*::Tn *codY*^{R222C}	This study
NE911	*ahpC*::Tn	(2)
SB527	*ahpC*::Tn *codY*^{R222C}	This study
NE1571	*ahpF*::Tn	(2)
NE1728	*bshA*::Tn	(2)
NE665	*perR*::Tn	(2)
NE390	*gltB*::Tn	(2)
SB529	*gltB*::Tn *codY*^{R222C}	This study
NE445	*umuC*::Tn	(2)
SB530	*umuC*::Tn *codY*^{R222C}	This study
NE563	*gpxA2*::Tn	(2)
SB531	*gpxA2*::Tn *codY*^{R222C}	This study
NE1730	*bsaA*::Tn	(2)
SB532	*bsaA*::Tn *codY*^{R222C}	This study
NE1803	RS04260::Tn	(2)
NE1332	*tpx*::Tn	(2)
NE1932	*sodA*::Tn	(2)
NE1224	*sodM*::Tn	(2)
NE122	*adhC*::Tn	(2)
SB533	*adhC*::Tn *codY*^{R222C}	This study
NE1538	*crtO*::Tn	(2)
SB534	*crtO*::Tn *codY*^{R222C}	This study
NE1692	*cidA*::Tn	(2)
SB536	*cidA*::Tn *codY*^{R222C}	This study
NE935	*cidB*::Tn	(2)
SB537	*cidB*::Tn *codY*^{R222C}	This study
NE564	*cidC*::Tn	(2)
SB538	*cidC*::Tn *codY*^{R222C}	This study
NE1466	*cidR*::Tn	(2)
SB539	*cidR*::Tn *codY*^{R222C}	This study
Escherichia coli		
DC10B	Cloning *E. coli* DC10B, *dam*/*dcm*-deficient	(4)
SB192	Cloning *E. coli* IM08B with USA300-pattern restriction modification	(5)

**Plasmids**		
pIMAY*	Allelic exchange vector	(6)
pIMAY*-codY^{R222C}	Allelic exchange vector for construction of the *codY** allele	This study
SB209	pKM16; fluorescent reporter	(7)
SB540	pKM16 backbone expressing *katA* from its native promoter	This study
SB541	pKM16 backbone expressing *ahpCF* from its native promoter	This study
SB542	pKM16 backbone expressing *umuC* from its native promoter	This study
SB543	pKM16 backbone expressing *pxpBCA* from its native promoter	This study
SB544	pKM16 backbone expressing *adhC* from its native promoter	This study

Supplemental Table 3. Oligonucleotides used in this study.

Primer Name	Description	Primer Sequence*
Sa035	Forward amplification of *codY* adding an EcoRI site with homologous sequence to pIMAY* for Gibson assembly	GGTATCGATAAGCTTGATATC**GAATTC**CGACAAAGTTGCGACGAATA
Sa036	Reverse amplification of *codY* adding a NotI site with homologous sequence to pIMAY* for Gibson assembly	TGGAGCTCCACCGCGGTG**GCGGCCGC**ATCACGAATTCCGCCTAAGA
Sa037	Forward pIMAY*-*codY* integration primers	AAGAAAGTTGAGCGAGAA
Sa038	Reverse pIMAY*-*codY* integration primers	AATTGCATTACGCTCTTT
JE2/qsrR/Up.F1	Upstream fragment for *qsrR* allelic exchange	TCACTAAAGGGAACAAAAGCTCTTTAACAACTAAATCATCTCCG
JE2/qsrR/Up.R1	Upstream fragment for *qsrR* allelic exchange	AGATGATGGAAGTATGTCCGCGTACTGCTAAATAATTACATGACG
IMAY*/qsrR/Up.F2	Vector linearization for *qsrR* allelic exchange	GCCTGCTTCAATTCTATCAGCGTCACAGGTATTTATTCGG
IMAY*/qsrR/Up.R2	Vector linearization for *qsrR* allelic exchange	GATGATTTAGTTGTTAAAGAGCTTTTGTTCCCTTTAGTGA
JE2/qsrR/Down.F3	Downstream fragment for *qsrR* allelic exchange	TGTAATTATTTAGCAGTACGCGGACATACTTCCATCATCTTC
JE2/qsrR/Down.R3	Downstream fragment for *qsrR* allelic exchange	CCGAATAAATACCTGTGACGCTGATAGAATTGAAGCAGGC
NTML/Buster2.R	Transposon primer (reverse)	GCCAACCTGTTACTAGACCG
NTML/Upstream2.F	Transposon primer (forward)	AAAGCATTGAACACCATAACCG
JE2/agrA/NTML.R1	Screening of *agrA* transposon insertion	TACACTGAATTACTGCCACG
JE2/cidA/NTML.R1	Screening of *cidA* transposon insertion	GAAAATGAAGTGAAATTTAGAGAGC
JE2/cidB/NTML.F1	Screening of *cidB* transposon insertion	TCATCCCAATTGAACTAAATGC
JE2/cidC/NTML.F1	Screening of *cidC* transposon insertion	GTTTAAGAATGGTCTTTCAGCA
JE2/cidR/NTML.R1	Screening of *cidR* transposon insertion	GAAATAGTTAGGATGATGTTAGTGG
JE2/katA/NTML.F1	Screening of *katA* transposon insertion	GCAGCTTGTTCAACATCC
JE2/ahpC/NTML.R1	Screening of *ahpC* transposon insertion	CATTACCTTCATCCATCTCG
JE2/ahpF/NTML.F1	Screening of *ahpF* transposon insertion	GCACGTATACCTGTCATTGC
JE2/perR/NTML.F1	Screening of *perR* transposon insertion	ATCATTGCGACAAGCAGG
JE2/gltB/NTML.F1	Screening of *gltB* transposon insertion	CTTGAAATGTTGCGACGC

JE2/gpxA2/NTML.F1	Screening of *gpxA2* transposon insertion	TCATTAGAACCTGGTTGTCG
JE2/bshA/NTML.F1	Screening of *bshA* transposon insertion	GATCATTCACTCCAAGGTGC
JE2/tpx/NTML.F1	Screening of *tpx* transposon insertion	ATAACATTCAAAGGTGGACC
JE2/umuC/NTML.F1	Screening of *umuC* transposon insertion	GTTGTTGCAGATACTAAGCG
JE2/RS04260/NTML.R1	Screening of *RS04260* transposon insertion	TTTATGGATTGACCCAATCG
JE2/crtO/NTML.R1	Screening of *crtO* transposon insertion	TCTCTAACATGAGAGTATTGGC
JE2/adhC/NTML.R1	Screening of *adhC* transposon insertion	ATACCTCCTAAACCAACAACCGC
pKM16/Dn/Linear.F1	Linearization of pKM16 excluding *sarA_dsRed*	CGGTTATCCACAGAATCAGG
pKM16/Up/Linear.R1	Linearization of pKM16 excluding *sarA_dsRed*	AAAGATCCTAACGAAAAGCG
JE2/umuC/Up.F1	*umuC* upstream primer for pKM_umuC	CGCTTTTCGTTAGGATCTTTCGATTGGCAACATCCAAACC
JE2/umuC/Dn.R1	*umuC* downstream primer for pKM_umuC	CCTGATTCTGTGGATAACCGAAACCCTACTGACCGAGAAC
JE2/katA/Up.F1	*katA* upstream primer for pKM_umuC	CGCTTTTCGTTAGGATCTTTTAAAATGTTGCCAACTCTCC
JE2/katA/Dn.R1	*katA* downstream primer for pKM_umuC	CCTGATTCTGTGGATAACCGCATAAACTGCTCAACTACGC
JE2/ahpCF/Up.F1	*ahpCF* upstream primer for pKM_umuC	CGCTTTTCGTTAGGATCTTTATCTTCTCATCGTCGATACC
JE2/ahpCF/Dn.R1	*ahpCF* downstream primer for pKM_umuC	CCTGATTCTGTGGATAACCGAAGCATTATCGCACATCTCG
JE2/pxpA/Up.F1	*pxpA* upstream primer for pKM_umuC	CCTGATTCTGTGGATAACCGTTCTCCCCATTTTTTTAGCC
JE2/pxpA/Dn.R1	*pxpA* downstream primer for pKM_umuC	GAAAAAATCGATGCGAGTTGATTTGAATTG
JE2/pxpBC/Up.F1	*pxpBC* upstream primer for pKM_umuC	CAACTCGCATCGATTTTTTCAATATTGATTTTACAAATCC
JE2/pxpBC/Dn.R1	*pxpBC* downstream primer for pKM_umuC	CGCTTTTCGTTAGGATCTTTGAATCACCAATGGCTAAAGC
JE2/adhC/Up.F1	*adhC* upstream primer for pKM_umuC	CGCTTTTCGTTAGGATCTTTAGTTTCATAATCCCACTCCC
JE2/adhC/Dn.R1	*adhC* downstream primer for pKM_umuC	CCTGATTCTGTGGATAACCGGAGCATCCTTCACTTTTGCG

***Bold indicates the restriction cut site.**

**Underlined sequences indicate regions of homology for Gibson assembly.

SUPPLEMENTARY DATA FILES

Supplementary Data File 01: A list of all mutations observed in each sequenced evolved isolate.

Supplementary Data File 02: The full edgeR analysis, differentially expressed genes, and enriched PANTHER pathways for each RNA-seq comparison.

Supplementary Data File 03: A list of genes comprising each pathway shown in the legend of the volcano plots.

Supplementary Data File 04: CodY sequences of *S. aureus* isolates shown by position.

Supplementary Data File 05: Scripts used for RNA-seq alignment and edgeR analysis.

Supplementary Data File 06: Raw processed data files from kallisto in tsv format.

REFERENCES

1. Miller LG, Rieg G, Bayer AS, Spellberg B. 2005. Necrotizing Fasciitis Caused by Community-Associated Methicillin-Resistant *Staphylococcus aureus* in Los Angeles. The New England Journal of Medicine 9.

2. Fey PD, Endres JL, Yajjala V, Widhelm TJ, Boissy RJ, Bose JL, Bayles KW. 2013. A Genetic Resource for Rapid and Comprehensive Phenotype Screening of Nonessential *Staphylococcus aureus* Genes. mBio 4:e00537-12.

3. Iordanescu S, Surdeanu M. 1976. Two Restriction and Modification Systems in *Staphylococcus aureus* NCTC8325. Journal of General Microbiology 96:277–281.

4. Monk IR, Shah IM, Xu M, Tan M-W, Foster TJ. 2012. Transforming the Untransformable: Application of Direct Transformation To Manipulate Genetically *Staphylococcus aureus* and *Staphylococcus epidermidis*. mBio 3:e00277-11.

5. Monk IR, Tree JJ, Howden BP, Stinear TP, Foster TJ. 2015. Complete Bypass of Restriction Systems for Major *Staphylococcus aureus* Lineages. mBio 6:e00308-15.

6. Schuster CF, Howard SA, Gründling A. 2019. Use of the counter selectable marker PheS* for genome engineering in *Staphylococcus aureus*. Microbiology 165:572–584.

7. Mlynek KD, Bulock LL, Stone CJ, Curran LJ, Sadykov MR, Bayles KW, Brinsmade SR. 2020. Genetic and Biochemical Analysis of CodY-Mediated Cell Aggregation in *Staphylococcus aureus* Reveals an Interaction between Extracellular DNA and Polysaccharide in the Extracellular Matrix. J Bacteriol 202:e00593-19, /jb/202/8/JB.00593-19.atom.
